# Risk for severe refractory *Mycoplasma pneumoniae* pneumonia complicated with bronchitis obliterans among children

**DOI:** 10.1097/MD.0000000000045664

**Published:** 2025-11-07

**Authors:** Zhonghua Hu, Chengchao Fang

**Affiliations:** a Department of Pediatrics, Linping Branch the Second Affiliated Hospital of Zhejiang University, Hangzhou, China.

**Keywords:** bronchitis obliterans, children, severe refractory *Mycoplasma pneumoniae* pneumonia

## Abstract

To investigate the independent risk factors for severe refractory MP pneumonia (SRMPP) complicated by bronchitis obliterans in children. A retrospective analysis was conducted on the clinical data of children with SRMPP admitted to the Department of Pediatrics from October 2022 to October 2024 in Linping branch the Second Affiliated Hospital of Zhejiang University. Differences in clinical characteristics between the 2 groups were compared. Multivariate logistic regression identified independent risk factors for SRMPP complicated with bronchitis obliterans, and receiver operating characteristic curve analysis evaluated. A total of 120 children with SRMPP were included, multivariate logistic regression analysis revealed that elevated D-dimer (D-D) levels (OR: 2.46, 95% CI: 1.41–4.81), elevated ferritin levels (OR: 2.93, 95% CI: 1.68–5.90), and the plastic mucus plugs (OR: 6.32, 95% CI: 4.32–9.64) were independent risk factors for SRMPP complicated with bronchitis obliterans (*P* < .01), while high serum albumin levels (OR: 0.82, 95% CI: 0.64–0.92) served as an independent protective factor (*P* = .01). Receiver operating characteristic curve analysis demonstrated that the areas under the curve for predicting bronchitis obliterans were 0.68 for elevated D-D, 0.66 for elevated ferritin, 0.72 for decreased albumin, and 0.70 for plastic mucus plugs. Elevated D-D, ferritin, and plastic mucus plugs are bronchitis obliterans risk factors in SRMPP children, high albumin is protective.

## 1. Introduction

*Mycoplasma pneumoniae* (MP) is an atypical pathogen and a common causative agent of community-acquired pneumonia. It can induce diseases of varying severity, ranging from mild upper respiratory tract infections to severe necrotizing pneumonia.^[1]^ While MP infections are typically mild, recent years have witnessed a rising incidence of MP infections, leading pediatricians to encounter an increasing number of cases of severe MP pneumonia (SMPP) and refractory MP pneumonia.^[2]^

In some children, MP infection may result in bronchitis obliterans, which can further progress to atelectasis and bronchiectasis.^[3]^ These complications manifest as persistent cough, dyspnea, wheezing, and other clinical symptoms, significantly impairing the affected children’s daily lives and academic performance. Early identification of high-risk severe refractory MP pneumonia (SRMPP) patients who may develop bronchitis obliterans and prompt clinical intervention are crucial for reducing its occurrence.

This study retrospectively analyzed the clinical characteristics and laboratory findings of SRMPP patients to identify predictive factors for bronchitis obliterans. The aim is to facilitate early recognition of high-risk populations and minimize long-term sequelae of SRMPP.

## 2. Subjects and methods

### 2.1. Study population

This retrospective study analyzed clinical data from pediatric inpatients diagnosed with SRMPP between October 2022 and October 2024 in Linping branch the Second Affiliated Hospital of Zhejiang University. This study was approved by the medical ethics committee of the Linping Branch of the Second Affiliated Hospital of Zhejiang University. Because of the retrospective nature of this study, the requirement for informed consent was waived. Inclusion criteria: age 1 to 14 years; diagnosis of SRMPP was based on the 2015 Chinese Expert Consensus on the Diagnosis and Treatment of MP pneumonia (MPP) in Children and the 2023 Chinese Guidelines for the Management of Pediatric MPP.^[1,3]^ Exclusion criteria: coinfection with other pathogens; underlying conditions such as asthma, immunodeficiency, or cardiopulmonary vascular malformations; and incomplete clinical records. Coinfection with other pathogens was excluded via: respiratory multiplex PCR (targeting influenza A/B, RSV, and adenovirus); blood cultures and systemic PCR where clinically indicated.

### 2.2. Methods

#### 2.2.1. Data collection

Demographic and clinical data included age, sex, body mass index, allergy history, fever duration, peak temperature, and pulmonary signs. Laboratory parameters assessed within 24 hours of admission included: Complete blood count: white blood cell count, neutrophil percentage (NEUT%), and platelet count. Inflammatory markers: C-reactive protein, procalcitonin, and interleukin-10. Coagulation and biochemistry: D-dimer (D-D), ferritin, albumin (ALB), alanine aminotransferase (ALT), lactate dehydrogenase, and creatine kinase-MB. Respiratory pathogen PCR panel: Influenza A/B, parainfluenza virus, respiratory syncytial virus, adenovirus, Mycoplasma, and Chlamydia.

#### 2.2.2. Bronchoscopy

All SRMPP patients underwent bronchoscopy following standardized protocols.^[4,5]^ Endoscopic evaluations included: mucosal changes, secretion characteristics, and presence of plastic mucus plugs (defined as dendritic casts extracted via suction or forceps).

Therapeutic interventions: bronchoalveolar lavage, removal of endobronchial debris, and recanalization of membranous obliteration using biopsy forceps.

#### 2.2.3. Diagnostic criteria for bronchitis obliterans and grouping

Diagnosis required either of the following^[3]^: Bronchoscopic evidence: obliteration of distal subsegmental bronchi ± proximal dilatation during convalescence (total disease course ≥ 1 month).^[3,6]^ Radiologic evidence: persistent atelectasis on chest imaging for > 6 months with minimal change over 3 months. Patients were stratified into: obliterated group and non-obliterated group.

### 2.3. Statistical analysis

Data were analyzed using SPSS 26.0 (IBM Corporation, Chicago). Normally distributed continuous variables: Mean ± SD; compared via independent *t*-tests. Non-normal data: Median (P25–P75); analyzed with Wilcoxon rank-sum tests. Categorical variables: Counts (%); assessed by χ² or Fisher exact tests. Logistic regression identified independent risk factors for bronchitis obliterans. Receiver operating characteristic curves evaluated biomarker utility. The multivariate model was constructed using backward stepwise logistic regression. Variables with *P* < .10 in univariate analyses were initially included, excluding correlated parameters (variance inflation factor ≥ 5). Final predictors were selected based on statistical significance (*P* < .05) and clinical relevance, with adjustment for age and sex.

## 3. Results

### 3.1. Comparison of clinical characteristics between groups

The study included 120 children with SRMPP (60 males and 60 females) with a median age of 6.0 years (4.0–8.0). The bronchial obliteration group (n = 40) had a median age of 6.5 years (5.0–8.0), while the non-obliteration group (n = 80) had a median age of 5.5 years (4.0–7.0). Although no significant difference was observed in age distribution between groups (*P* > .05), the proportion of children aged ≥ 5 years was significantly higher in the obliteration group (*P* < .05).

Compared with the non-obliteration group, the obliteration group showed significantly higher prevalence rates of allergy history, asthma, and hypoxemia (*P* < .05). Bronchoscopic examination revealed significantly higher proportions of mucosal erosion or necrosis and plastic mucus plugs in the obliteration group (*P* < .05; Table 1).

**Table 1:** Comparison of clinical features between occluded group and non-occluded group.

	Obliteration (n = 40)	Non-obliteration (n = 80)	*t*/x^2^	*P*
Sex (male, %)	18 (45)	42 (52.5)	0.06	.83
Age, yr	6.5 (5–8)	6 (4–7)	4.7	.03
BMI	15.5 ± 1.6	15.3 ± 1.2	0.81	.42
Atopic dermatitis n (%)	1 (2.5)	2 (2.5)	0.00	1.00
Asthma n (%)	8 (20)	2 (2.5)	5.22	.02
Allergic rhinitis n (%)	1 (2.5)	2 (2.5)	0.00	1.00
Fever time/d	9 (8–11)	9 (8–10)	0.10	.92
SpO2 < 93% n (%)	13 (32.5)	10 (12.5)	5.11	.02
Mucosal erosion or necrosis (%)	28 (70)	10 (28.6)	15.40	.00
Plastic mucus plugs (%)	29 (72.5)	0	21.46	.00

BMI = body mass index.

### 3.2. Comparison of laboratory findings

The obliteration group demonstrated significantly higher levels of C-reactive protein, D-D, ferritin, and interleukin-10, along with significantly lower albumin levels compared to the non-obliteration group (*P* < .05; Table 2).

**Table 2:** Comparison of laboratory test results between occluded group and non-occluded group.

	Obliteration (n = 40)	Non-obliteration (n = 80)	*t*/z	*P*
WBC 10^9^/L	8.2 ± 1.6	7.6 ± 2.2	1.59	.15
NEU %	65.6 ± 10.5	67.2 ± 6.8	1.08	.30
PLT 10^9^/L	265.5 ± 64.5	253 ± 48.4	1.80	.07
CRP mg/L	46.4 ± 12.5	32.1 ± 10.4	4.68	.01
PCT	0.2 (0.05–0.25)	0.2 (0.1–0.3)	0.65	.52
LDH u/L	245.2 ± 64.5	240.6 ± 48.2	0.42	.66
D-D	2.2 (1.7–3.4)	1.6 (1.1–2.3)	3.6	.01
ALB	30.5 ± 5.4	42.5 ± 4.2	16.4	.01
Ferritin	254.5 ± 36.8	116.5 ± 42.1	4.25	.01
IL-10	5.6 ± 1.2	4.8 ± 1.1	3.45	.01
CK-MB	3.7 (1.8–4.6)	3.7 (1.9–4.8)	0.06	.95

ALB = albumin, CK-MB = creatine kinase-MB, CRP = C-reactive protein, D-D = D-dimer, IL-10 = interleukin-10, LDH = lactate dehydrogenase, PCT = procalcitonin, PLT = platelet count, WBC = white blood cell count.

### 3.3. Predictive factors and their diagnostic value for bronchitis obliterans in SRMPP patients

Multivariate logistic regression analysis identified elevated D-D, increased ferritin, and presence of plastic mucus plugs as independent risk factors for bronchitis obliterans in SRMPP patients (*P* < .05), while higher serum albumin level served as a protective factor (Table 3).

**Table 3:** Multivariate regression analysis of risk factors for SRMPP.

Factors	Cut value	SE	95% CI	OR	*P*
ALB	0.37	0.47	0.64–0.92	0.82	.01
D-D	0.05	0.02	1.41–4.81	2.46	.01
Plastic mucus plugs	0.09	0.06	4.32–9.64	6.32	.01
Ferritin	0.23	0.26	1.68–5.90	2.93	.01

ALB = albumin, D-D = D-dimer, SRMPP = severe refractory MP pneumonia.

**Figure 1.**
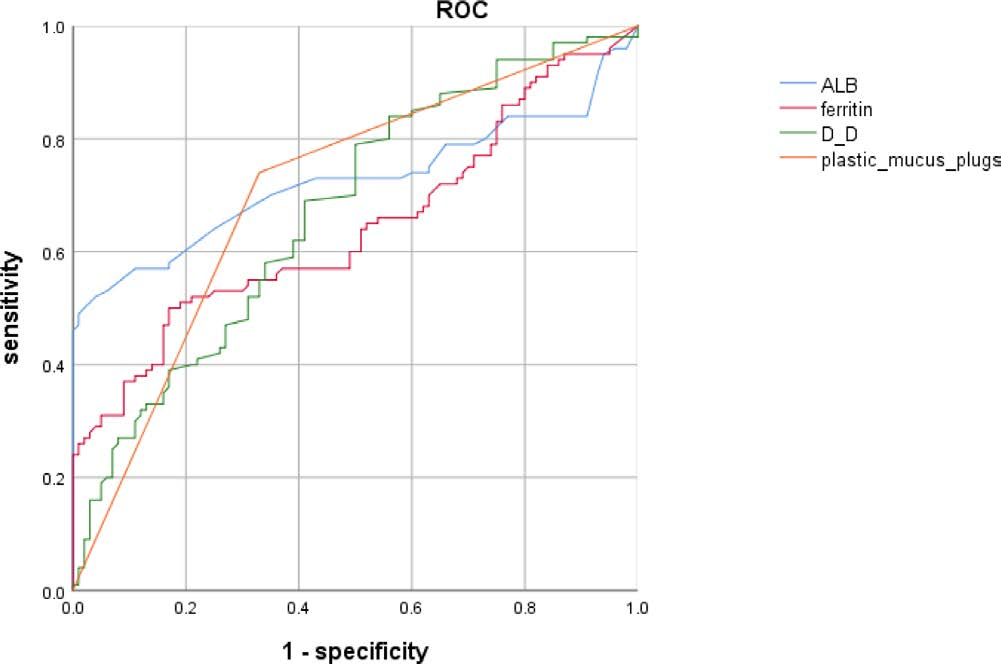
Predictive value analysis of each factor in children with SRMPP complicated with bronchitis obliterans by ROC curve. ROC = receiver operating characteristic, SRMPP = severe refractory MP pneumonia.

Receiver operating characteristic curve analysis revealed that the area under the curve values for predicting bronchitis obliterans were 0.68 for D-D elevation, 0.66 for ferritin elevation, 0.72 for albumin reduction, and 0.70 for plastic mucus plugs (Fig. 1).

## 4. Discussion

SRMPP stands out for its fast-paced disease progression and a wide range of clinical symptoms that are often difficult to predict. As the condition advances, it frequently gives rise to life-threatening pulmonary complications – including necrotizing pneumonia, pulmonary embolism, obliterative bronchitis, and atelectasis – and each of these complications puts the health of affected children in serious jeopardy.^[7]^ From a clinical standpoint, the number of children developing obliterative bronchitis has been on the rise in recent years, making this condition one of the most commonly seen long-term pulmonary consequences in pediatric patients.^[8]^ What is more, major medical centers across China have successively reported a growing number of cases related to obliterative bronchitis.^[9]^

It is essential to distinguish obliterative bronchitis from bronchiolitis obliterans, as the 2 conditions differ substantially in both pathological traits and clinical signs. Pathologically, obliterative bronchitis is mainly characterized by the growth of inflammatory granulomatous tissue and the formation of fibrosis in small-to-medium bronchi that contain cartilage. These pathological changes eventually lead to the onset of chronic airflow obstruction syndrome. When it comes to clinical symptoms, the most obvious sign of obliterative bronchitis is atelectasis or the collapse of an entire lung lobe.^[10]^ During electronic bronchoscopy examinations, medical teams often observe that the far-end parts of subsegmental and sub-subsegmental bronchi are blocked in patients with this condition. In some cases, additional irregularities – such as the widening of proximal bronchi, the shrinking of bronchial mucosa, and the exposure of cartilage rings – can also be detected.^[3]^

Up to now, the exact way in which Mycoplasma infection brings about obliterative bronchitis has not been fully worked out. Even so, existing research findings point to the possibility that inflammatory reactions and immune system activities may play a part in triggering this pathological process.^[11]^ In the current study, 36.3% of SRMPP patients developed obliterative bronchitis – a rate that is similar to what has been reported in other relevant studies.^[10]^ One possible reason for this similarity is that our hospital, which focuses on treating severe pediatric cases, has included a larger number of children with severe, hard-to-treat SRMPP who were transferred from other hospitals. That said, we cannot ignore the fact that a small sample size and potential biases in the information collected might also have influenced the incidence rate observed in this study. Beyond that, multiple research projects have indicated that children who are 5 years old or older face a greater chance of developing obliterative bronchitis after being diagnosed with SRMPP. The most widely accepted explanation for this trend is that the immune systems of children aged ≥ 5 years are more developed than those of younger children. While this more mature immune system allows them to fight off pathogen attacks more effectively, it can also lead to the overstimulation of immune-inflammatory pathways. This overactive immune response then causes the bronchial mucosa to produce more secretions than normal, and these secretions have a high concentration of mucin. Such a situation creates ideal conditions for the formation of mucus plugs; these plugs then block the airways, which in turn makes the existing inflammatory damage even worse.^[12,13]^ As time goes on, this gradual process damages the normal structure of the airways, eventually resulting in complications like obliterative bronchitis, bronchiectasis, and atelectasis. Other studies have also supported this idea, noting that being ≥ 5 years old is a key risk factor for the formation of mucus plugs in children with MPP.^[14]^

Atopy describes a physical state where the body is prone to developing conditions like atopic dermatitis, allergic rhinitis, and asthma when exposed to external triggers. This state involves the abnormal differentiation of CD4⁺ T helper type 2 cells and the overproduction of immunoglobulin E.^[15]^ Earlier studies have suggested that children with atopy are more likely to get infected with Mycoplasma, and such infections can cause a significant increase in immunoglobulin E levels – especially during the acute phase of the infection or when the child is in an allergic state. This finding suggests that Mycoplasma might play a role in triggering type I hypersensitivity reactions in MPP, and that atopy could be linked to how SRMPP becomes.^[16,17]^ In line with these previous studies, the current research found that a much higher percentage of children in the obliterative bronchitis group had atopy (e.g., asthma) compared to those not in this group. The proposed reason for this is that after being infected with Mycoplasma, children with atopy are more likely to have type I hypersensitivity reactions. These reactions release substances like enzymes, histamines, and cytokines, which then cause the airways to become overly reactive, produce too much mucus, and suffer structural damage. All of these changes make it more likely for the child to develop SRMPP and, eventually, obliterative bronchitis.

Albumin is a type of negative acute-phase protein, meaning its levels drop quickly when the body is dealing with an infection, under stress, or experiencing liver dysfunction. Because of this, albumin levels can be used to assess both the nutritional status of the body and the severity of inflammatory reactions.^[18]^ Recent research has found connections between prealbumin and albumin levels on one hand, and the severity of a disease and its outcome on the other.^[19]^ For example, a retrospective study showed that ICU patients with low albumin levels (hypoalbuminemia) had a higher chance of dying, which means albumin can be used as a marker to predict how a patient’s condition will progress.^[20]^ Similarly, the current study found that low albumin levels were linked to worse outcomes in SRMPP patients, and that hypoalbuminemia is an independent risk factor for the development of obliterative bronchitis.

When the body is infected with Mycoplasma, the pathogen releases toxins and causes the overproduction of inflammatory cytokines. Both of these factors damage the cells that line blood vessels (vascular endothelium) and activate the body’s blood-clotting system. In children with severe MPP, ongoing fevers, lack of oxygen (hypoxia), and acidosis (an imbalance in the body’s acid levels) make this process even worse, leading to higher levels of D-D in the blood plasma. Since D-D levels reflect how severe an inflammatory reaction is and the risk of blood clots forming, this is often used as a biomarker to track how a disease is progressing.^[21,22]^ Studies have reported that in children with MPP, there is a positive relationship between D-D levels and the severity of the disease.^[23]^ In cases of refractory MPP, high D-D levels are a sign of excessive inflammation and damage to the vascular endothelium.^[24]^ Similarly, the current study identified that D-D level is an independent risk factor for obliterative bronchitis in SRMPP patients.

### 4.1. Limitations

While our study identified significant independent risk factors for bronchitis obliterans in SRMPP, the moderate area under the curve values of these parameters suggest that no single biomarker is sufficient for definitive prediction. This may reflect the multifactorial pathogenesis of bronchitis obliterans, where interactions between host immune responses, pathogen virulence, and environmental factors collectively contribute to disease progression. Future studies should explore combinatorial models integrating clinical, imaging, and multi-omics data to improve predictive accuracy.

## 5. Conclusion

For children with SRMPP exhibiting serum albumin, plasma D-D levels, ferritin levels and the formation of plastic mucus plugs, clinicians should remain vigilant for the potential development of bronchitis obliterans.

## Acknowledgments

The authors thank all of the subjects who participated in this study

## Author contributions

**Conceptualization:** Chengchao Fang.

**Data curation:** Zhonghua Hu, Chengchao Fang.

**Formal analysis:** Zhonghua Hu.

**Investigation:** Zhonghua Hu.

**Methodology:** Zhonghua Hu.

**Project administration:** Zhonghua Hu.

**Resources:** Zhonghua Hu.

**Validation:** Chengchao Fang.

**Writing – original draft:** Chengchao Fang.

**Writing – review & editing:** Zhonghua Hu.
